# PlasForest: a homology-based random forest classifier for plasmid detection in genomic datasets

**DOI:** 10.1186/s12859-021-04270-w

**Published:** 2021-06-26

**Authors:** Léa Pradier, Tazzio Tissot, Anna-Sophie Fiston-Lavier, Stéphanie Bedhomme

**Affiliations:** 1 Centre d’Ecologie Fonctionnelle et Evolutive, CNRS, Université de Montpellier, Université Paul Valéry Montpellier 3, Ecole Pratique des Hautes Etudes, Institut de Recherche Pour le Développement, 34000 Montpellier, France; 2 Genomics, Bioinformatics and Evolution. Departament de Genètica i Microbiologia, Universitat Autònoma de Barcelona, 08193 Cerdanyola del Vallès, Spain; 3 Centre de Recerca Matemàtica, 08193 Cerdanyola del Vallès, Spain; 4 Institut des Sciences de l’Evolution de Montpellier (ISE-M), Equipe Evolution, Vecteurs, Adaptation et Symbiose, UMR 5554, CNRS-Université Montpellier, 34090 Montpellier Cedex 05, France

**Keywords:** Plasmid identification, Homology, Random forest classifier, Genomic datasets

## Abstract

**Background:**

Plasmids are mobile genetic elements that often carry accessory genes, and are vectors for horizontal transfer between bacterial genomes. Plasmid detection in large genomic datasets is crucial to analyze their spread and quantify their role in bacteria adaptation and particularly in antibiotic resistance propagation. Bioinformatics methods have been developed to detect plasmids. However, they suffer from low sensitivity (i.e*.*, most plasmids remain undetected) or low precision (i.e., these methods identify chromosomes as plasmids), and are overall not adapted to identify plasmids in whole genomes that are not fully assembled (contigs and scaffolds).

**Results:**

We developed PlasForest, a homology-based random forest classifier identifying bacterial plasmid sequences in partially assembled genomes. Without knowing the taxonomical origin of the samples, PlasForest identifies contigs as plasmids or chromosomes with a F1 score of 0.950. Notably, it can detect 77.4% of plasmid contigs below 1 kb with 2.8% of false positives and 99.9% of plasmid contigs over 50 kb with 2.2% of false positives.

**Conclusions:**

PlasForest outperforms other currently available tools on genomic datasets by being both sensitive and precise. The performance of PlasForest on metagenomic assemblies are currently well below those of other k-mer-based methods, and we discuss how homology-based approaches could improve plasmid detection in such datasets.

**Supplementary Information:**

The online version contains supplementary material available at 10.1186/s12859-021-04270-w.

## Background

Plasmids are extra-chromosomal fragments of DNA that replicate autonomously in the host cell. They often carry genes that can provide a benefit under specific environmental conditions [1]. These mobile genetic elements remain a major biological concern for health and agriculture policies due to their ability to accumulate and spread resistance genes. Indeed, the frequency of plasmids, and of the resistance genes they carry, can increase quickly in populations thanks to their high mobility both within hosts (through chromosomal integration) and between hosts (through horizontal gene transfer, hereafter HGT). Due to their high mobility and the function of the genes they carry, plasmids have a great ecological importance in many bacterial communities.

Until recently, the identification and isolation of these mobile genetic elements was limited to a narrow subsample of the bacteria diversity. Most past studies have only focused on specific species-plasmid associations of medical or agronomic interest [2, 3]. On the one hand, the plasmid characteristics determining their role in resistance propagation were established by phenotypic approaches, which require the focal strain to grow on culture media. These approaches could assess the ability of genetic elements to transfer small conjugative plasmids carrying selectable markers into a new recipient, i.e. transfer, replication and expression of selectable markers [4], but were useless for non conjugative plasmids. On the other hand, PCR-based detection methods [5] could detect the number of copies for specific plasmid sequences, but did not allow to understand the ecological characteristics of mobile genetic elements, as for example their host range.

The fast development of sequencing technologies and reduction of sequencing costs led to the rapid increase of available genomic and metagenomic data sets. This material contains a vast amount of information on plasmid diversity, plasmid host-range, resistance conferred to specific host taxa, etc. that could allow to better understand the circulation and spread of plasmids and the genes they carry. Accessing this information requires new tools to process partially assembled datasets to identify plasmids sequences.

Well-defined plasmids can be identified through homology search (e.g., PlasmidFinder [6], PLACNET, PLACNETw [7]). These programs basically look for similarities between a query sequence and a local database. Query and subject sequences are usually quite long (at least several hundreds of bases), so the probability of finding similarities by chance (and thus to wrongly identify a sequence as plasmid) is very low. Homology search is usually very precise and reliable. However, current data limitations can substantially decrease the sensitivity of this method. Indeed, it requires an exhaustive database composed of a wide variety of genomics data, enough to cover many taxa, and even if this is the case, rare plasmids may not be identified. In particular, the range of taxa in which a plasmid can be replicated and maintained (i.e., its host range [5]) can greatly vary: some being restricted to a few close species, and others consisting in a wide range of taxa across the phylogeny. Thus, plasmids with a narrow host range (especially when only present in uncultivated species) could be nearly impossible to identify through homology.

In the absence of an exhaustive database, an accurate identification method requires to define broader associations with plasmids. This can be achieved by the reconstruction of plasmids through homology-based clustering (PLACNET, PLACNETw [7], MOB-recon [8]): the query sequences which are homologous to the same reference plasmid sequences are likely to be part of the same plasmids. This approach can be successful in two cases: (1) if the generated graphs are manually pruned by an expert user (PLACNET, PLACNETw [7], but this strongly impedes the possibility to apply this method to large datasets) and (2) if the query sequences are also compared to complementary databases (e.g. insertion sequences and repeated elements, MOB-recon [8]).

Other algorithms identify plasmids in draft genomes and metagenomes without relying on homologies, but rather on k-mers frequencies: PlasFlow [9]; cBar [10]; PlasmidSeeker [11]; PlasClass [12]; PPR-Meta [13]). Scoring sequences based on their k-mers frequencies is quick, automated, and adaptable to various data, and may result in valuable predictions (e.g.*,* more than 95% of correct predictions for PlasFlow [9]). However, the frequencies of k-mers cannot be estimated with precision in a short contig, even when short k-mers (e.g.*,* 7-mers for PlasFlow [9]) are used. For this reason, k-mer-based methods systematically rule out contigs below 1 kb. To get around such limitations, more recent approaches rather rely on mathematical models of sequence composition, such as one-hot matrices (PPR-Meta, [13]), thereby achieving better performances on short sequences.

Overall, the current plasmid identification methods are limited, in either precision or sensitivity. These limitations are not problematic for certain applications: for example, k-mer-based methods are well adapted to detect plasmid sequences in metagenomics samples containing many uncultivable species and homology-based approaches are well suited if users focus only on a few plasmids of interest. Yet, no tool is currently available to classify, with both high precision and sensitivity, a large data set of sequences as plasmid or chromosome. Such a tool would be very useful for example to monitor the spread of a gene family (e.g.*,* antibiotic resistance genes) using partially assembled genomic datasets. Indeed, 90% of available assemblies on public repositories (up to 100% for species with few sequenced genomes) are partially assembled genomes (contigs or scaffolds). It is thus not possible to rely on the size of the genomic elements and their content in specific sequences (as ribosomal RNA genes or origin of replications) to classify them as chromosome or plasmids and an accurate plasmid identification method is required.

Here, we present PlasForest, a new tool for classifying contigs in partially assembled genomic datasets as plasmid or chromosome. Contrary to classical homology-based tools, our method does not attempt to assign query contigs to specific subject sequences, but rather to sort contigs in plasmid and chromosome sequences through a machine learning algorithm fed with parameters extracted from an homology search against an exhaustive plasmid database, as well as other variables (e.g.*,* contig size and %G + C content). PlasForest can discriminate plasmids from chromosome sequences with an overall F1 score of 0.950 for any bacterial contig in genomic datasets. In particular, PlasForest is able to detect up to 77.4% of plasmid contigs under 1 kb with only 2.8% false-positives and up to 99.9% of plasmid contigs over 50 kb with less than 2.2% false-positives. Compared to other currently available tools, PlasForest has a significantly better capacity to correctly identify plasmids from chromosomes in partially assembled genomes. We implemented this tool in a user-friendly pipeline able to identify plasmids in large datasets in a reasonable amount of time.

## Results

PlasForest is a tool that allows to assign contigs in genomic datasets to plasmid or chromosome by using a random forest classifier on variables extracted from a homology search. We tested the sensitivity of its performances to various changes in the training process, and we provide a comparison with other classical plasmid identification tools on large genomic datasets.

### Pipeline description

**Fig. 1:**
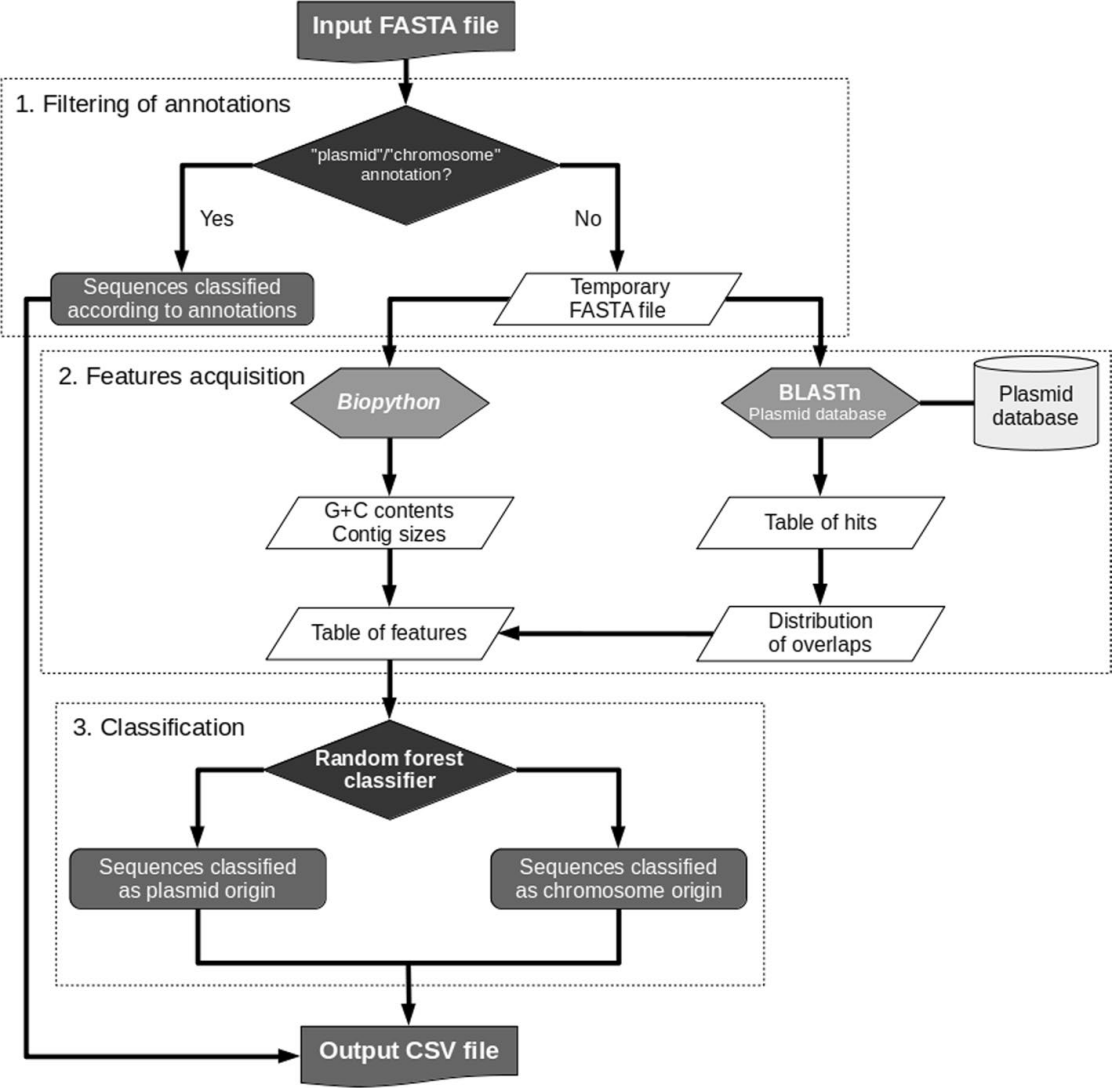
General method of classification implemented in PlasForest

**Fig. 2:**
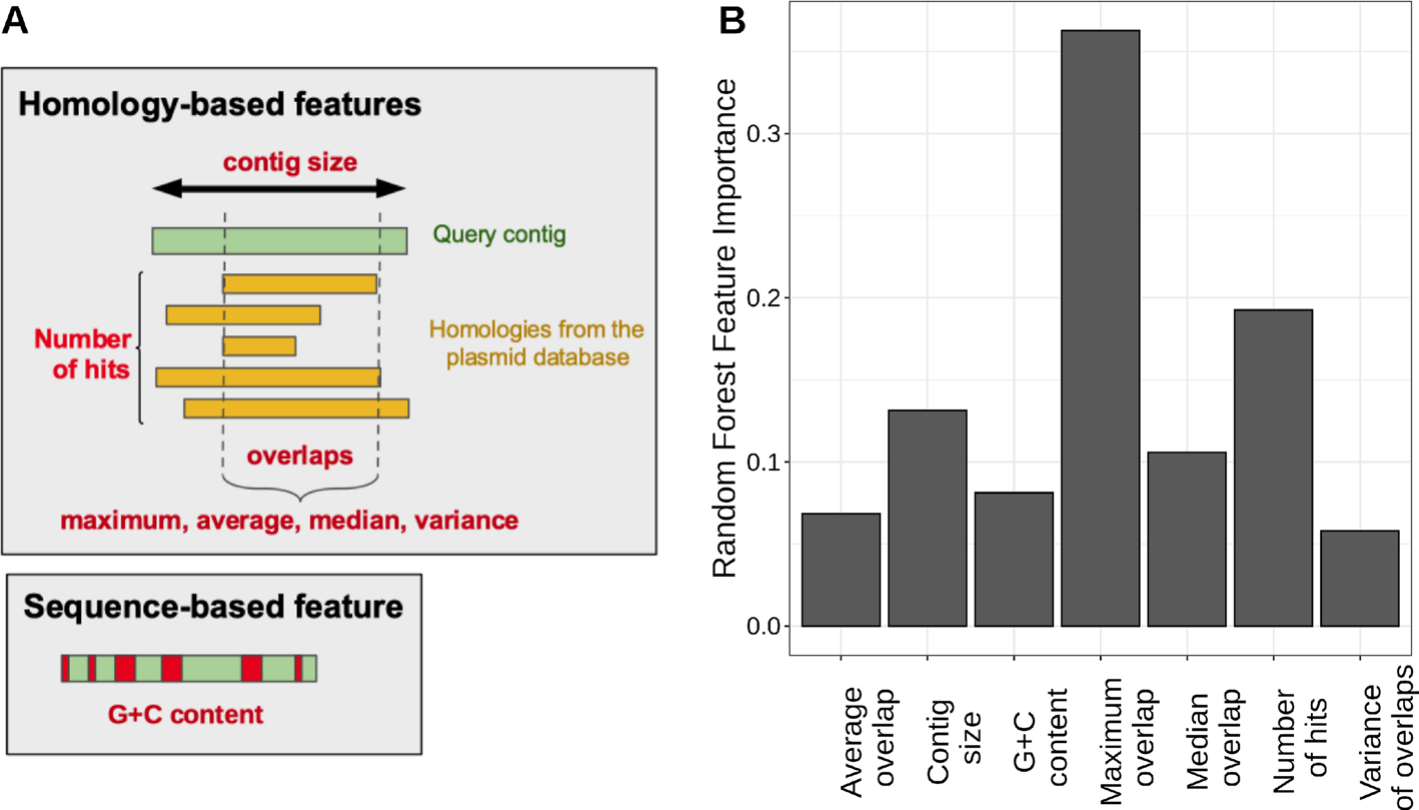
Chosen features and their importances in the classification process. **A** Schematic representation of the features extracted from contigs, including homology-based features (number of hits, maximum overlap, average overlap, median overlap, variance of overlaps, contig size) and sequence-based feature (G + C content). **B** Impurity-based feature importance computed with *scikit-learn* library for the seven features kept in the classifier

A pipeline executing all analyses required for PlasForest was encoded in Python 3.6. This pipeline performs all the different steps described in Fig. 1. First, it filters query contigs by their annotations, to avoid re-identification of sequences already described as chromosome or plasmid (step 1). If the user wants to re-assign contigs with Plasforest, this step can be skipped with option *-r*. Feature acquisition (step 2) consists of submitting the filtered sequences to BLASTn against a local copy of the plasmid database, and calculation of the overlaps between query and subject sequences. Features are then computed for each query contig on its sequence and the distribution of overlaps. The pipeline thus extracts seven features: average overlap, contig size, G + C content, max overlap, median overlap, number of hits, variance overlap (see Fig. 2A). The classification (step 3) passes features to the random forest classifier, which outputs the predicted identification for each query contig. The output can also include the best hits that were found in the plasmid database with option *-b* and/or the features used for classification with option *-f*. Progression of the pipeline can be displayed if run with option *-v*. Predictions can be split to several threads with the multithreading option *-threads* and/or to several batches with the option *-size_of_batch.*

We tested runtime and memory usage for the pipeline on a subset of 1000 contigs randomly drawn from the COMGENOME dataset (see Methods). For a single thread, PlasForest takes on average 490 s and uses a maximum of 2.3 gigabytes of memory (compared to respectively 300 s and 600 megabytes for PlasClass—a k-mer-based tool—on the same dataset). The evolution of these performances with the number of threads is displayed in Additional file 1: Fig. S1.

### Reliability of the classification method

PlasForest was trained on artificial draft genomes, created by randomly cutting completely assembled genomes into contigs following a size distribution observed in actual draft genomes (Additional file 4: Table S1), that we called balanced training set (Additional file 5: Table S2A). When run on the testing set (Additional file 6: Table S2B) with all seven features (Fig. 2A), PlasForest presented a good ability to discriminate between plasmid and chromosome sequences, with both a high sensitivity (92.7%) and a high precision (97.3%). We tested the sensitivity of PlasForest qualitative changes by evaluating the change in performance when adding or removing features in the training of the classifier and when resampling the training dataset and the plasmid database.

The classifier was trained several times on the balanced training set, by feeding it with different combinations of features. Predictions of the classifiers were made on the testing set. The classification showing the highest MCC (Matthews correlation coefficient) was the one using all the features considered (*average overlap, contig size*, *G* + *C content*, *max overlap*, *median overlap*, *number of hits, variance overlap*), and the three variables that showed the highest feature importance were the *maximal overlap*, the *number of hits*, and *contig size* (Fig. 2B).

**Fig. 3:**
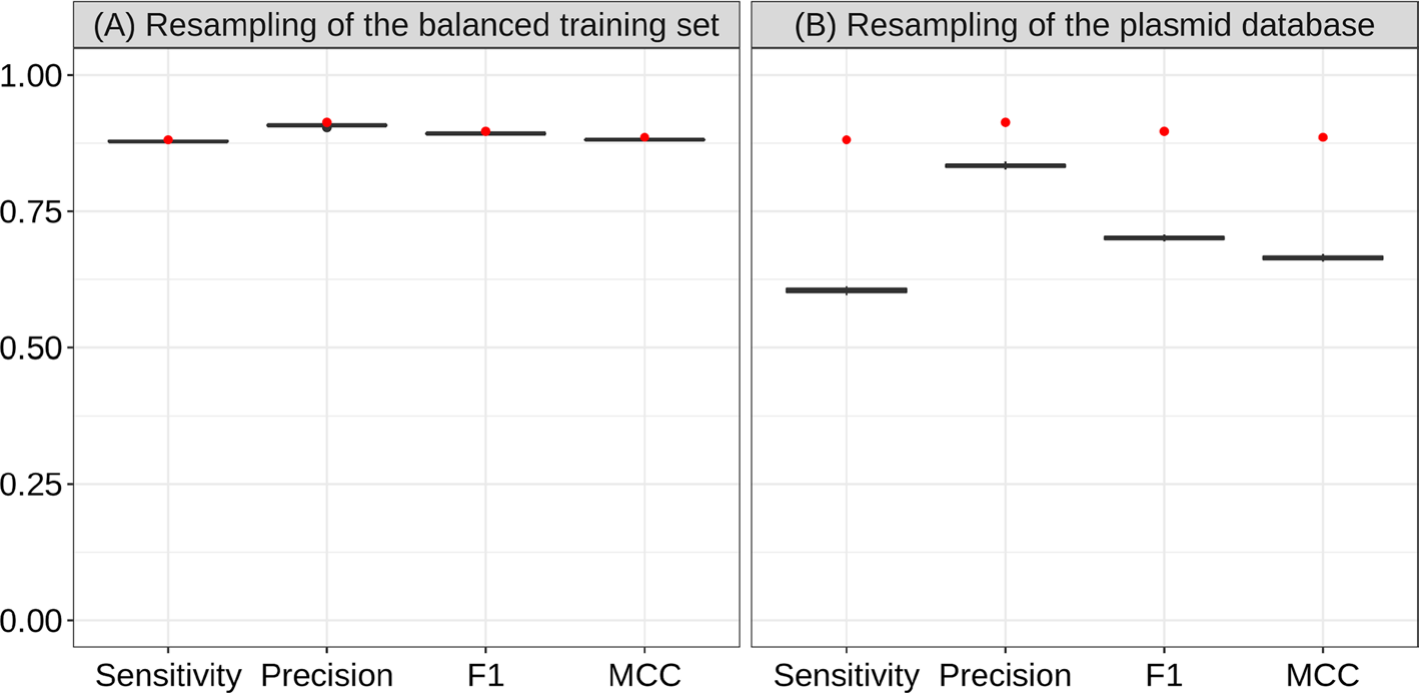
Sensitivity of PlasForest to resampling. **A** Performances after 50 resampling of the balanced training set. **B** Performances after 50 resampling of the plasmid database. The initial performances of PlasForest on the testing set are displayed with red dots. The distribution of performances for PlasForest when resampling 50 times either the plasmid database or the balanced training set are displayed in grey boxes

Bootstrapping the training set and the plasmid database showed that the performance of PlasForest presents a very low sensitivity to the resampling of the training set (see Fig. 3A). However, the performance of PlasForest decreases substantially when the composition of the plasmid database is resampled (see Fig. 3B). Removing sequences from the plasmid database can therefore significantly diminish the ability of plasForest to correctly assign contigs.

### Benchmark of plasmid identification methods

**Fig. 4:**
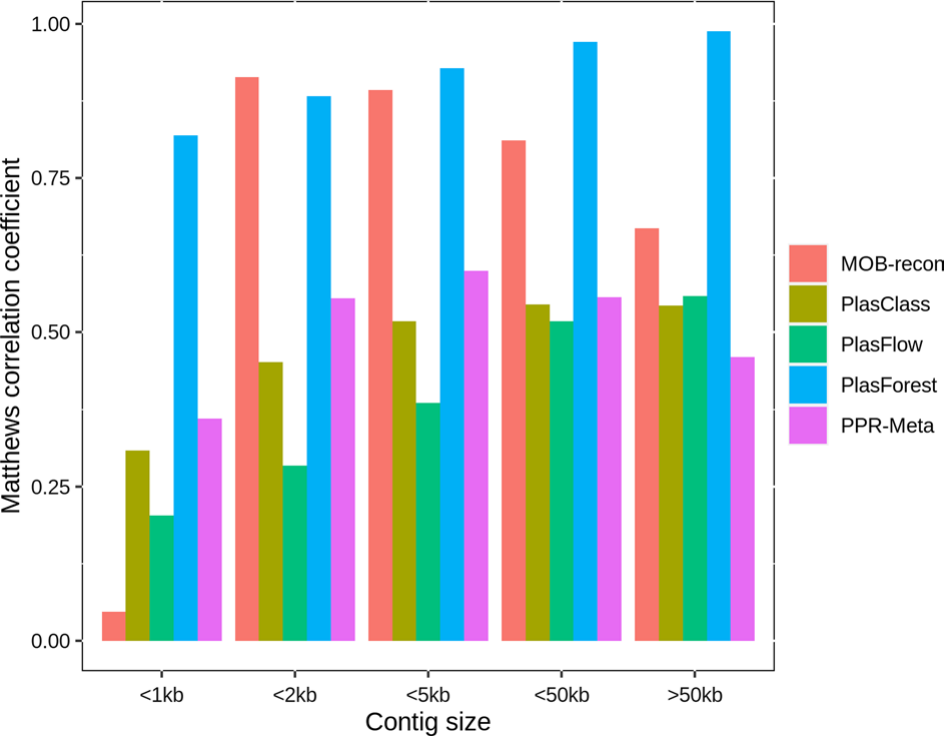
Compared performances of PlasForest and 4 other plasmid identification methods on the testing set

We compared the predictions of PlasForest on our testing dataset (see Table 1 and Fig. 4), with those of three classification methods based on genomic signatures (PlasFlow [9], PPR-Meta [13], and PlasClass [12]) and one classical homology-based classification method (MOB-recon [8]). PlasmidSeeker [11] was not included in our analysis, because it requires to know the species from which the genome comes and therefore cannot be used on a wide scale.

**Table 1:** Compared performances of PlasForest and 4 other plasmid identification softwares on the testing dataset

Contig size	Index	PlasFlow	PlasClass	Mob-Recon	PPR-Meta	PlasForest
[50 bp, 1 kb)	Sensitivity	0.850	0.672	0.003	0.585	0.774
	Precision	0.373	0.473	1	0.550	0.972
	F1	0.518	0.555	0.006	0.567	0.862
	MCC	0.203	0.309	0.047	0.361	0.819
[1 kb, 2 kb)	Sensitivity	0.832	0.749	0.888	0.763	0.826
	Precision	0.294	0.451	0.973	0.558	0.986
	F1	0.434	0.563	0.928	0.644	0.899
	MCC	0.284	0.451	0.914	0.555	0.883
[2 kb, 5 kb)	Sensitivity	0.871	0.787	0.870	0.805	0.903
	Precision	0.308	0.464	0.946	0.552	0.975
	F1	0.455	0.584	0.907	0.655	0.938
	MCC	0.386	0.518	0.893	0.599	0.929
[5 kb, 50 kb)	Sensitivity	0.899	0.816	0.729	0.889	0.976
	Precision	0.366	0.432	0.932	0.413	0.971
	F1	0.520	0.565	0.818	0.564	0.973
	MCC	0.518	0.545	0.811	0.557	0.971
[50 kb, + ∞)	Sensitivity	0.912	0.761	0.500	0.944	0.999
	Precision	0.374	0.423	0.919	0.259	0.978
	F1	0.530	0.544	0.648	0.406	0.988
	MCC	0.559	0.543	0.669	0.459	0.988
Overall	Sensitivity	0.883	0.785	0.670	0.831	0.927
	Precision	0.348	0.443	0.939	0.431	0.973
	F1	0.499	0.566	0.782	0.568	0.950
	MCC	0.483	0.531	0.775	0.540	0.945

PlasForest is overall the most sensitive classifier, being able to predict 92.7% of plasmid contigs. However, for sequences below 2 kb, PlasForest is less sensitive than PlasFlow with down to 77.4% of plasmids correctly predicted. PlasForest had the highest precision, followed by MOB-recon (with respectively 2.7% and 6.1% of sequences incorrectly predicted as plasmids). The precision of PlasForest is lower than that of MOB-recon only for contigs below 1 kb (respectively 2.8% and 0% of sequences incorrectly predicted as plasmids). On these two indices, PlasForest is therefore globally the best software, though other classifiers can have higher sensitivity or precision for short contigs. However, PlasFlow suffers from low precision, especially below 5 kb (up to 70.6% of false positives between 1 and 2 kb), and the sensitivity of MOB-recon can be very low (down to 0.3% of true positives below 1 kb, down to 50% above 50 kb). PlasForest therefore outperforms all other programs on the composite indices as well, with the highest MCC (globally 0.945, and even 0.988 for contigs above 50 kb) and the highest F1 score (globally 0.950, and even 0.988 for contigs above 50 kb), except for contigs between 1 and 2 kb where it is outperformed by MOB-recon. Even for very short contigs (under 1 kb) for which k-mers-based methods usually have poor results, PlasForest remains a reliable classifier, with MCC = 0.819 and F1 = 0.862 (respectively MCC = 0.361 and F1 = 0.567 for PPR-Meta, the second-best classifier for these sizes).

### Applicability of PlasForest to other datasets

We tested further the ability of PlasForest to predict contigs of plasmid origin on three additional datasets. We also compared the results obtained with PlasForest to the results obtained with the four other programs on these three datasets. The COMGENOME dataset gathers artificial contigs, randomly cut with the same size distribution as the testing set, but drawn from complete genomic assemblies that were released after the training and testing sets were constituted (Additional file 7: Table S3). The CONTIG dataset contains partially assembled genomes which were present neither in the training nor in the testing datasets (Additional file 2: List S1), and the METAGENOME dataset contains genomes that were partially assembled from metagenomic short reads (extracted from the datasets of [14] and [15], Additional file 3: List S2). Contrary to the COMGENOME dataset, the genomes used to create the CONTIG and the METAGENOME dataset were not annotated, so we could not measure the accuracy of the predictions of the different programs on these datasets.

On the COMGENOME dataset (see Table 2), PlasForest performances are reduced compared to its performances on the testing set, notably in sensitivity (only 54.3% true positives, compared to 85.7% true positives detected by PlasFlow). Yet, PlasForest remains as precise, with only 11.3% false positives (compared to 9.3% false positives detected by MOB-recon). Overall, on this dataset, it remains the best classifier, with MCC = 0.663 and F1 = 0.674 (respectively MCC = 0.522 for MOB-recon, and F1 = 0.538 for PlasFlow).

**Table 2:** Compared predictions of PlasForest and 4 other plasmid identification softwares on the COMGENOME dataset

Index	PlasFlow	PlasClass	Mob-Recon	PPR-Meta	PlasForest
Sensitivity	0.857	0.723	0.338	0.665	0.543
Precision	0.392	0.460	0.907	0.414	0.887
F1	0.538	0.563	0.493	0.511	0.674
MCC	0.493	0.500	0.522	0.436	0.663

**Fig. 5:**
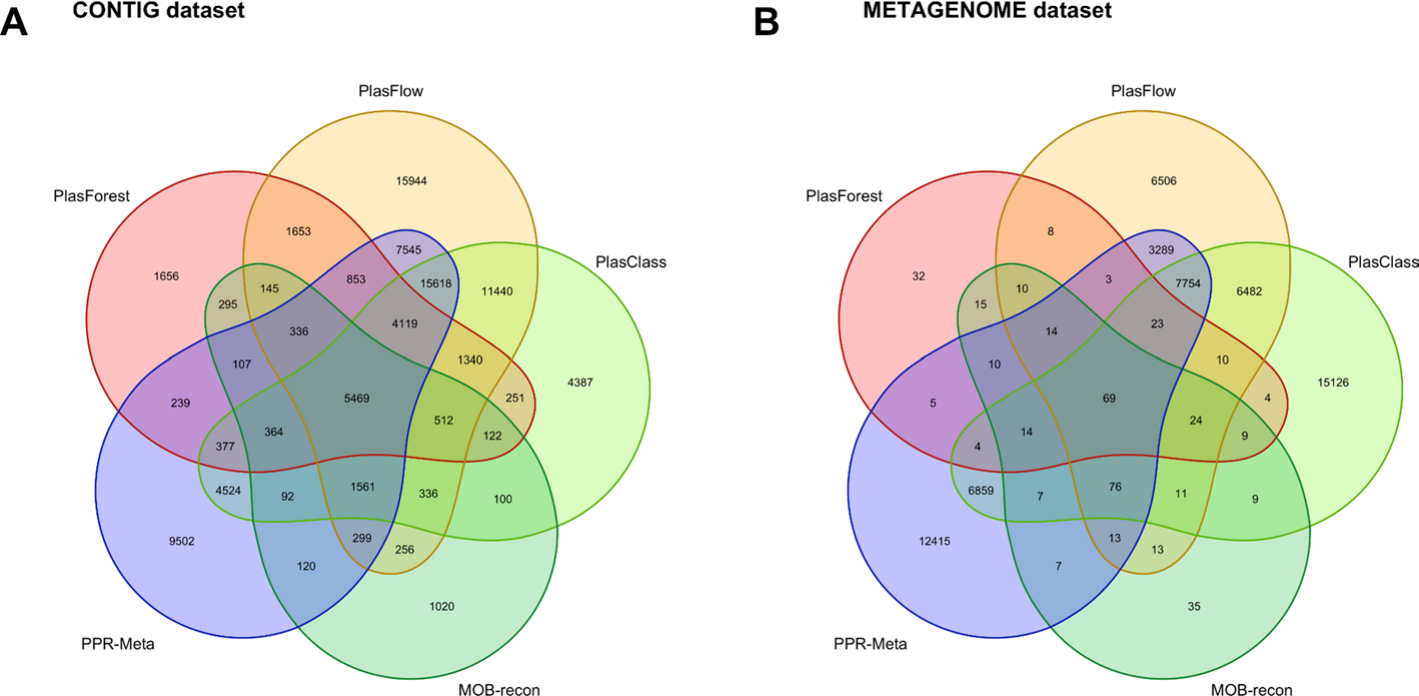
Agreement of plasmid identification for PlasForest and 4 other plasmid identification methods on the CONTIG and METAGENOME datasets. **A** Number of contigs identified as plasmids in the CONTIG dataset. **B** Number of contigs identified as plasmids in the METAGENOME dataset. The CONTIG dataset gathers 151,634 contigs collected from 1328 partially assembled genomes. The METAGENOME dataset gathers 143,663 contigs collected from 1000 partially assembled genomes drawn from metagenomic datasets

On the CONTIG dataset (see Fig. 5A), PlasForest and PlasFlow agree to predict that 14,427 contigs originate from plasmids (80.9% of plasmids predicted by PlasForest). However, 52,999 contigs that PlasFlow detects as plasmids are identified as chromosomes by PlasForest (39.6% of chromosomes predicted by PlasForest). PlasFlow is thus the plasmid identification method whose predictions resemble less those of PlasForest (Cohen’s κ = 0.187). In general, plasmid identification methods based on k-mers disagree with the predictions of PlasForest on this dataset (Cohen’s κ = 0.232 between PlasClass and PlasForest, and Cohen’s κ = 0.205 between PPR-Meta and PlasForest). On the contrary, though PlasForest and MOB-recon only agree to predict that 7350 contigs originate from plasmids (41.2% of plasmids predicted by PlasForest), only 3783 contigs that MOB-recon detects as plasmids are identified as chromosomes by PlasForest (2.8% of chromosomes predicted by PlasForest). Predictions of MOB-recon and PlasForest thus globally agree substantially (Cohen’s κ = 0.459).

On the METAGENOME dataset (see Fig. 5B), PlasForest and MOB-recon globally identify very few contigs as plasmids (respectively 0.18% and 0.24% of contigs). On the contrary, PlasFlow, PlasClass and PPR-Meta all predict a much higher number of contigs as plasmids (up to 25.4% of contigs for PlasClass). However, it is important to note that these three methods have a low level of agreement regarding which contigs are predicted to be plasmids: PPR-Meta and PlasClass agree on only 48.4% of contigs predicted as plasmids by PPR-Meta (Cohen’s κ = 0.271), while this number drops to 36.8% when comparing PPR-Meta and PlasFlow (Cohen’s κ = 0.269).

## Discussion

PlasForest is a homology-based approach, combined with machine learning, which detects plasmids in contig and scaffold genomes. Its operating principle is to seek homologies between query contigs and a large plasmid database, and then to assign a plasmid/chromosome identity to queries with a random forest classifier.

Here we showed that PlasForest is able to deal with large datasets without prior knowledge of the taxonomic background, and that it identifies plasmids with both a high sensitivity and a high precision in unassembled genomes. All the plasmid identification software tested had either a higher false negative error rate or a higher false positive error rate than PlasForest, especially on very short contigs (below 1 kb). PlasForest did not always have the lowest individual error rates, but it optimized the tradeoff between sensitivity and precision, and as a consequence had the highest values of the composite indices F1 and MCC for the vast majority of the size classes. In terms of biological material, PlasForest can be used on draft genomes (in form of contigs and scaffolds) as well as on assembled genomes. PlasForest has been trained and tested on artificial draft genomes and has good performance on them (overall MCC = 0.945; and even MCC = 0.819 on contigs below 1 kb).

K-mer-based approaches were traditionally the most reliable plasmid identification methods. However, the number of possible k-mers increases exponentially with k and accurately estimating the frequency of all possible k-mers requires large contigs even for small values of k. For example, estimating the frequency of all 256 possible 4-mers in a contig requires thousands of bases, while the size of the shortest known plasmids is below 1 kb [16]. Thus, PlasForest outperforms those methods, especially on short contigs, because the quality of the genomic signature is much less dependent on contig size with homology-based features than with k-mers. K-mer-based approaches are thus valuable only when used on long contigs or in some very specific contexts: for example, PlasmidSeeker [11] achieves up to 100% sensitivity and 99.8% specificity in whole genome sequencing reads by ruling out as chromosomal the k-mers which are shared with a complete, reference assembly. However, the use of PlasmidSeeker on broad diverse datasets is practically infeasible: it requires to know the species from which the genome comes from and that at least one genome has been assembled for this species [11].

The high precision of PlasForest is inherited from its homology-seeking basis. Though homology-based approaches may traditionally show little sensitivity, due to their inability to detect unrelated plasmids, the performances of PlasForest and MOB-recon have significantly improved plasmid identification. One of the reasons why PlasForest is as sensitive as (or even more than) k-mer-based approaches is that it aggregates measures of homologies in a classifier. Thus, it not only considers the presence of homologies, but it also measures the quality and diversity of these homologies and this improves the accuracy of the identification process.

Many bacterial species remain uncultivable but an increasing number of metagenomics tools have allowed access to genomic data in environmental samples without the necessity of obtaining pure cultures. Other identification tools specially designed for such data (e.g.*,* PlasFlow [9]) offer the opportunity of taxonomic assignment of the sequence identified as belonging to plasmids. During the development of PlasForest, we addressed the classification of contigs from genomic (and not metagenomic) data into plasmids or chromosomes. The pipeline of PlasForest offers the possibility to identify which plasmids from the database have the strongest homology with the query. It should be noted though, that the taxonomic assignment can strongly depend on (1) the plasmid host range and (2) horizontal gene transfer events that are yet massively undetermined in bacteria. Any attempt (from PlasForest or from any other method) of taxonomic assignment for broad host range plasmids is thus at least imprecise and can sometimes be impossible [17].

Finally, PlasForest is the best alternative for plasmid identification in genomic assemblies. However, as PlasForest mostly relies on homologies with a plasmid database, the vast majority of plasmids it is able to detect are related to those of its database. It thus showed reduced performances when resampling its database or when testing it on the COMGENOME dataset, which included genomes published after the construction of the training and test sets. Unrelated plasmids, especially with narrow host ranges or when the host is uncultivable, are difficult to detect through this method so far. Thus, in its current state, PlasForest cannot be applied to detect plasmids in metagenomic assemblies. Both PlasForest and MOB-recon, two identification methods that were initially designed for genomic assemblies, detected very few contigs as plasmids in the METAGENOME dataset. Though it is very difficult to predict the actual frequency of contigs originating from plasmids, it is e.g. 12.7% in the COMGENOME dataset. It is thus far higher than 0.2% so PlasForest and MOB-recon most probably have a very low sensitivity for metagenomic assemblies. However, we argue that investing in homology-based approaches for plasmid detection in metagenomic assemblies is crucial. Indeed, methods based on detection of k-mers probably allow to detect a much higher number of plasmid contigs in unknown species, but they also have a high false-positive rate: they detected up to 25% of plasmid contigs in the METAGENOME dataset. We also showed that their predictions are in high disagreement with one another, therefore rendering them hard to rely on. That is why assembling methods such as metaPlasmidSPAdes [18] also rely on homology search, but these methods only apply to newly sequenced datasets and in general substantially increase the running time of assembly. As novel methods to detect plasmids in metagenomes need to rely on homology search, new datasets will be required. Some sequences from assembled plasmidomes (some being already available on MG-RAST database [19]) could be incorporated in the plasmid database of PlasForest. But most importantly, when plasmids can be mechanically separated from chromosomes in metagenomic samples [20], both chromosomes and plasmids should be sequenced: PlasForest could then be trained on annotated metagenomic datasets. As these datasets become more and more available, it will be possible to update PlasForest such that it will become able to detect plasmids in metagenomes.

## Conclusions

In its current state, among all softwares tested, PlasForest is the best identification method for plasmids in contigs and scaffolds. As shown with the COMGENOME dataset, PlasForest still outcompetes other methods on recently released genomes. We released PlasForest as a user-friendly pipeline, including the trained classifier and the plasmid database, directly available on GitHub (https://github.com/leaemiliepradier/PlasForest). Further releases (at least on a half-year basis) will include a plasmid database updated with the new plasmid sequences submitted to public repositories, and a new classifier trained on this database. This complemented database should be regularly trimmed, in order to keep it of reasonable size without decreasing the performance of PlasForest. Especially in order to identify plasmids in metagenomic data with better accuracy than k-mer-based approaches, plasmidomes should be included in the plasmid database and the classifier should also be trained on annotated metagenomic assemblies.

## Methods

The aim of PlasForest is to combine both the high precision of homology search with the broad sensitivity of signature-based classifiers in order to discriminate contigs of plasmid origins from contigs of chromosomes. We trained a classifier for which decision relies on the homology of sequences with a large database of plasmid sequences. We simulated contigs by randomly cutting assembled genomes, to construct both a dataset to train the classification algorithm (the training set) and a dataset to measure the classification performance (the testing set). We then compared the classification performance to other plasmid identification tools.

### Data collection

#### Plasmid database

All bacterial plasmid sequences were downloaded from the NCBI RefSeq Genomes FTP server (ftp://ftp.ncbi.nlm.nih.gov/genomes/refseq; September 1st, 2019). This database is composed of 36,450 sequences that we used as reference for homology seeking via BLAST tool [21] ⁠(e-value < 10^–3^).

#### Training and testing datasets

To train the classifier and measure its performance, we randomly sampled 10,152 bacterial genomes classified as 'complete', downloaded from the NCBI Refseq Genomes FTP server (ftp://ftp.ncbi.nlm.nih.gov/genomes/refseq).

**Fig. 6:**
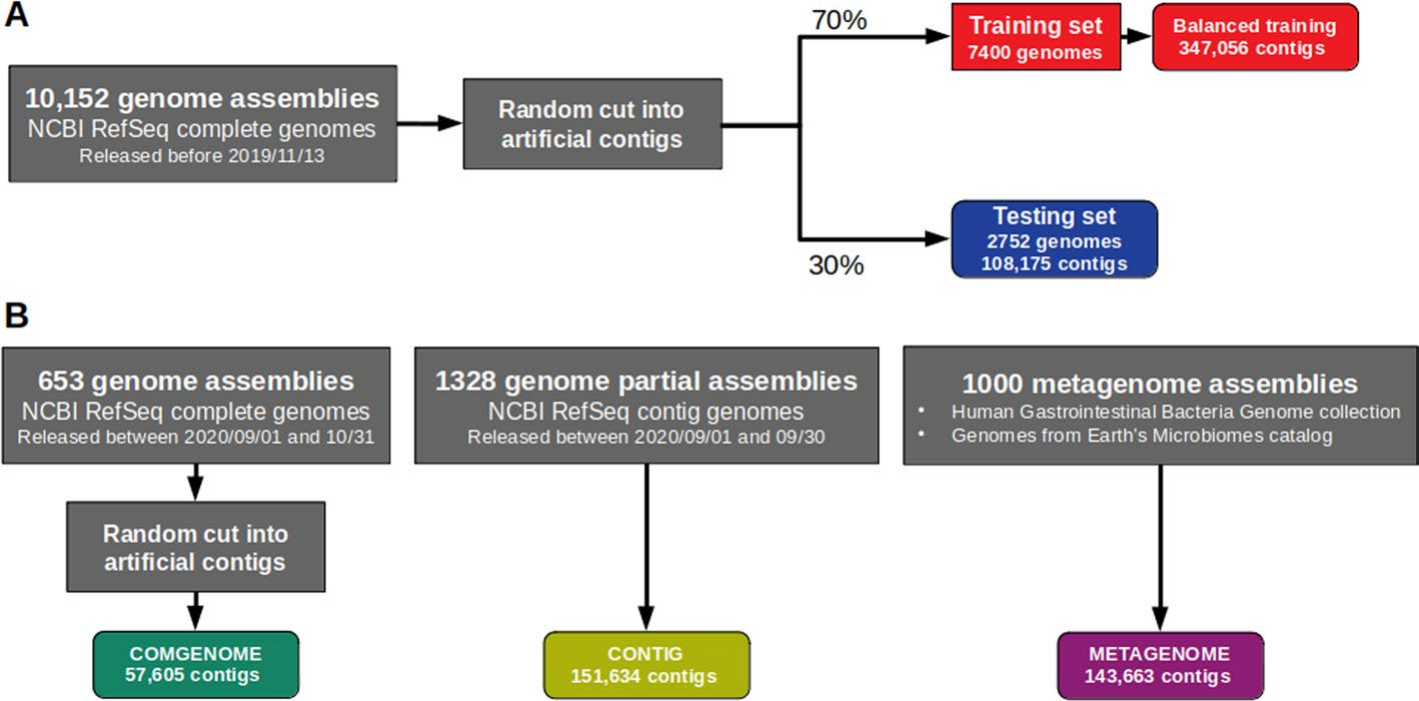
Datasets and application of a hold-out method for supervised learning. Schematic representation of the processes that allow to generate the datasets used to build PlasForest and to benchmark its performances. **A** 10,152 bacterial genomes from NCBI Refseq Genomes FTP server were randomly cut into contigs, and were distributed into the following datasets: the *(balanced) training set* contains 70% of the initial 10,152 genomes assemblies and it is used to train the random forest classifier; the *testing set* contains 30% of the genomes. **B** Other genome assemblies were drawn from more recent releases of NCBI Refseq Genomes FTP or from other sources to build the COMGENOME, CONTIG, and METAGENOME datasets. With the testing set, they are used to benchmark the performance of PlasForest compared to other plasmid identification methods

To mimic the sequence material on which PlasForest will be applied (contigs from unassembled whole genomes), the empirical distribution of contig sizes was established from more than 100,000 Refseq unassembled genomes (see Additional file 4: Table S1 for the chosen distribution). This distribution was then artificially recreated from complete genomes in the training and testing sets by cutting plasmids and chromosomes at random locations and keeping a defined number of each contig size in plasmids and chromosomes. Only contigs larger than 50 bp were kept, since most current sequencing approaches do not produce shorter reads [22]. We ended up with approximately 70% of the generated contigs (552,410 contigs coming from 7,400 genomes) to train PlasForest. Genome annotations were used to identify contigs as plasmids or chromosomes (see Fig. 6A). The remaining 30% of the generated contigs (108,175 contigs coming from 2,752 genomes) were used as a testing set. In these two datasets, plasmid contigs were not at the same frequencies for all the contig sizes (e.g., > 30% under 1 kb and 2% over 100 kb). This could have led to an artificial detection bias based on contig size (e.g., a better identification of small plasmid contigs). Thus, we split the initial training dataset into contig size categories (50 bp to 1 kb, 1 to 2 kb, 2 to 5 kb, 5 to 10 kb, 10 to 50 kb, 50 to 100 kb, and over 100 kb), and randomly removed plasmid or chromosome contigs from each category to keep the fraction of plasmid contigs constant (around 10%) across contig sizes. This new dataset is thereafter called *balanced training set* (Additional file 5: Table S2A). No manipulation of the testing set was performed (Additional file 7: Table S2B).

#### Use case datasets

We created three other datasets to validate the performance of our approach on several use cases (see Fig. 6B).

The CONTIG dataset was created by using all the 1328 bacterial genomes classified as 'contig' that were released on RefSeq between September 1st and 30th, 2020. This dataset gathered 151,634 contigs (see Additional file 2: List S1 for the list of genome identifiers).

The COMGENOME dataset was created by using all the 653 bacterial genomes classified as 'complete' that were released on RefSeq between September 1st and October 31st, 2020. These genomes were thus released after PlasForest was trained, and none of their sequences were used to create either the training and testing sets or the plasmid database. To simulate draft genome assemblies, the sequences from these genomes were randomly cut into contigs following the same size distribution as used to create the training and testing sets (Additional file 4: Table S1), but no correction was applied to the frequency of contigs of plasmid origin. This process resulted in 57,605 contigs (see Additional file 7: Table S3 for the list of artificial contigs).

The METAGENOME dataset was created by using two subsamples of draft genomes assembled from metagenomic short reads. 500 genomes were randomly sampled from the Human Gastrointestinal Bacteria Genome collection [14], and 500 genomes from the Genomes from Earth’s Microbiomes catalog [15], thus bringing together 143,663 contigs (see Additional file: List S2 for the list of genome identifiers).

### Construction of PlasForest

#### Extraction of the features

All contigs were compared against the plasmid database using BLASTn [21]. Pair-alignments with homologous sequences (hereafter referred to as “hits”) were kept if their e-value was below 10^−3^. For each contig and homologous sequence, we computed *overlap* as the fraction of the query contig aligning to the homologous sequence hit. The G + C content of all contigs was computed with the function *SeqUtils.GC* from the Biopython library in Python 3.6 [23].

Our aim is not to assemble plasmids (or to assign contigs to precise replicons), but to identify contigs that originate from plasmids. This motivates for a distinct design from other homology-based approaches. By combining both homology search and measures of nucleotide composition, we aim to obtain a strong distinction between plasmids and chromosomes. We therefore selected features as follows. (1) ***Maximal overlap*** was measured among hits in the subject database, because we expect that query plasmid contigs should form longer alignments with sequences from the plasmid database than query chromosomes. (2) ***Contig size*** was included as short contigs align more often than large contigs with the subject database. (3) The ***number of hits***, the ***average overlap***, the ***median overlap***, and the ***variance of overlaps*** provide other parameters of the distribution of overlaps among hits, that may help distinguish between chromosomes and plasmids. Indeed, due to recombination events, one may expect that query chromosome contigs will align with subject plasmids, but more rarely than query plasmid contigs. (4) Finally, the ***G* + *C content*** was also included, as the nucleotide composition of plasmids are most often different from those of chromosomes [24]. This set of features used to train the classifier is schematically displayed in Fig. 2A.

#### Training of the classifier

We extracted the differences in the features of plasmid contigs and chromosome contigs thanks to a random forest classifier. This approach relies on a multitude of independent decision trees, which allows for a reduction of individual error [25]. The aim was therefore to build a model able to predict, from the extracted features, whether a contig comes from a plasmid or a chromosome. The random forest classifier algorithm was trained with the *RandomForestClassifier* function from *scikit-learn* library [26] in Python 3.6, using the seven features described above. The number of random decision trees was kept to 500, as out-of-bag error estimate (i.e., the internal error of individual decision trees during the training process) did not significantly decrease when using more trees. The global classification method of PlasForest is described in Fig. 1.

#### Sensitivity of the classifier

We tested the sensitivity of PlasForest (1) to the composition of the plasmid database and (2) to the composition of the balanced training set, by performing two independent bootstrap analyses. To assess the importance of the composition of the plasmid database, we resampled the plasmid database with 50 different seeds. We then computed new features for each contig of the balanced training set and testing set. A classifier was trained on the balanced training set for each resampled plasmid database, and its performance was measured on the testing set. To test the sensitivity to the composition of the balanced training set, we resampled the balanced training set 50 times, while the testing set did not change. We trained classifiers on the resulting balanced training sets, and measured their performances on the testing set.

### Measure of classification performances

#### Indices of binary classification performance

In order to measure the performance of our trained algorithm to correctly identify plasmid sequences and to compare its performance to other available tools, we computed indices derived from the confusion matrix that are commonly used in binary classifications. Sensitivity (sometimes indicated as recall) is the fraction of positive data (in our case, plasmid contigs) which has been correctly identified as positive and allows to measure the false negative error rate. Precision (also indicated as the positive predictive value) corresponds to the fraction of positive results that are actually true positives and allows to measure the false positive error rate. A good classifier should be able to optimize both sensitivity and precision i.e. in our case, identify as many plasmid contigs as possible without misidentifying chromosome contigs as plasmids. For this reason, we calculated “composite” indices that reflect the overall performance of the classifier. F1 score corresponds to the harmonic mean of sensitivity and precision: it therefore ranges from 0 (i.e.*,* either precision or sensitivity or both are null) to 1 (i.e.*,* there are neither false positives nor false negatives). F1 score does not take into account true negatives. We also calculated *Matthews Correlation Coefficient* (MCC). This metric corresponds to a correlation coefficient between the observed and the predicted classifications and is generally regarded as a balanced measure that can even be used if classes are of very different sizes [27]. Values range between + 1 for a perfect prediction, 0 for a random prediction, and -1 for a prediction in total disagreement with the observed data.

#### Comparison with other softwares

We ran 4 other plasmid identification softwares on the same datasets as PlasForest, and compared their predictions and performances to those of PlasForest. The version 1.1 of PlasFlow was downloaded from https://github.com/smaegol/PlasFlow⁠. Taxonomic assignments were not taken into account to assess the performance of the classification. To avoid the algorithm assigning sequences as “unclassified”, we used a threshold value of 0.5. MOB-suite was downloaded from https://github.com/phac-nml/mob-suite, and MOB-recon was run with default values. MOB-recon clustering algorithm requires draft genome assemblies as inputs, so contigs on which MOB-recon was tested were gathered by their genome of origin. PlasClass was downloaded from https://github.com/Shamir-Lab/PlasClass and the program was run with default values. The virtual machine version of PPR-Meta was downloaded from http://cqb.pku.edu.cn/ZhuLab/PPR_Meta, and the program was run in VirtualBox v. 5.2.42 with default values. When the algorithm assigned sequences as “phage”, they were considered as negative predictions.

## Supplementary Information

**Additional file 1.** **Figure S1:** Compared running time and memory use for PlasForest and PlasClass.

**Additional file 2.** **List S1:** Identifiers of contigs used to create the CONTIG dataset.

**Additional file 3.** **List S2:** Identifiers of contigs used to create the METAGENOME dataset.

**Additional file 4.** **Table S1:** Empirical distribution of contig sizes used to cut complete genomes into contigs.

**Additional file 5.** **Table S2A:** Identifiers and positions of contigs used to create the balanced training set.

**Additional file 6.** **Table S2B:** Identifiers and positions of contigs used to create the testing set.

**Additional file 7.** **Table S3:** Identifiers and positions of contigs used to create the COMGENOME dataset.

## Data Availability

PlasForest was released on GitHub (https://github.com/leaemiliepradier/PlasForest) and is available under GPLv3 license. The NCBI accession numbers of sequences used in this study can be retrieved in the Additional file 5: Table S2A for the balanced training set, Additional file 6: Table S2B for the testing set, Additional file 7: Table S3 for the COMGENOME dataset, and List S1 for the CONTIG dataset. The list of NCBI accession numbers of sequences used for the plasmid database is available on the PlasForest GitHub repository (https://github.com/leaemiliepradier/PlasForest/blob/master/list_ids.txt). The sequences used for the METAGENOME dataset can be retrieved from HGG collection (ftp://ftp.ebi.ac.uk/pub/databases/metagenomics/hgg_mags.tar.gz) and GEMs catalog (https://genome.jgi.doe.gov/portal/GEMs/GEMs.home.html), with accession numbers available in Additional file: List S2. All the sequence coordinates necessary to cut complete genomes into contigs (for the balanced training set, the testing set, and the COMGENOME dataset) are available in Additional file 5: Table S2A, Additional file 6: Table S2B and Additional file 7: Table S3.

## Abbreviations

HGT: Horizontal gene transfer
MCC: Matthews correlation coefficient

## References

[1] Elwell LP, Shipley PL (1980). Plasmid-mediated factors associated with virulence of bacteria to animals. Annu Rev Microbiol.

[2] Johnson TJ, Logue CM, Johnson JR, Kuskowski MA, Sherwood JS, Barnes HJ (2012). Associations between multidrug resistance, plasmid content, and virulence potential among extraintestinal pathogenic and commensal Escherichia coli from humans and poultry. Foodborne Pathog Dis.

[3] Poolkhet C, Chumsing S, Wajjwalku W, Minato C, Otsu Y, Takai S. Plasmid profiles and prevalence of intermediately virulent rhodococcus equi from pigs in Nakhonpathom Province, Thailand: Identification of a new variant of the 70-kb virulence plasmid, type 18. Vet Med Int. 2010;2010. 10.4061/2010/491624 PMC2860478 20445784

[4] Costa R, Götz M, Mrotzek N, Lottmann J, Berg G, Smalla K (2006). Effects of site and plant species on rhizosphere community structure as revealed by molecular analysis of microbial guilds. FEMS Microbiol Ecol.

[5] Heuer H, Binh CTT, Jechalke S, Kopmann C, Zimmerling U, Krögerrecklenfort E (2012). IncP-1ε plasmids are important vectors of antibiotic resistance genes in agricultural systems: diversification driven by class 1 integron gene cassettes. Front Microbiol.

[6] Carattoli A, Zankari E, García-Fernández A, Larsen MV, Lund O, Villa L (2014). In silico detection and typing of plasmids using plasmidfinder and plasmid multilocus sequence typing. Antimicrob Agents Chemother.

[7] Vielva L, De Toro M, Lanza VF, De La Cruz F (2017). PLACNETw: a web-based tool for plasmid reconstruction from bacterial genomes. Bioinformatics.

[8] Robertson J, Nash JHE (2018). MOB-suite: software tools for clustering, reconstruction and typing of plasmids from draft assemblies. Microb Genomics.

[9] Krawczyk PS, Lipinski L, Dziembowski A (2018). PlasFlow: predicting plasmid sequences in metagenomic data using genome signatures. Nucleic Acids Res.

[10] Zhou F, Xu Y (2010). cBar: A computer program to distinguish plasmid-derived from chromosome-derived sequence fragments in metagenomics data. Bioinformatics.

[11] Roosaare M, Puustusmaa M, Möls M, Vaher M, Remm M (2018). PlasmidSeeker: Identification of known plasmids from bacterial whole genome sequencing reads. PeerJ.

[12] Pellow D, Mizrahi I, Shamir R (2020). PlasClass improves plasmid sequence classification. PLOS Comput Biol.

[13] Fang Z, Tan J, Wu S, Li M, Xu C, Xie Z, et al. PPR-Meta: a tool for identifying phages and plasmids from metagenomic fragments using deep learning. 2019;8:1–14. 10.1093/gigascience/giz066. 10.1093/gigascience/giz066 PMC6586199 31220250

[14] Forster SC, Kumar N, Anonye BO, Almeida A, Viciani E, Stares MD (2019). A human gut bacterial genome and culture collection for improved metagenomic analyses. Nat Biotechnol.

[15] Nayfach S, Roux S, Seshadri R, Udwary D, Varghese N, Schulz F (2020). A genomic catalog of Earth’s microbiomes. Nat Biotechnol.

[16] Ciok A, Dziewit L, Grzesiak J, Budzik K, Gorniak D, Zdanowski MK (2016). Identification of miniature plasmids in psychrophilic Arctic bacteria of the genus Variovorax. FEMS Microbiol Ecol.

[17] Suzuki H, Yano H, Brown CJ, Top EM (2010). Predicting plasmid promiscuity based on genomic signature. J Bacteriol.

[18] Antipov D, Raiko M, Lapidus A, Pevzner PA (2019). Plasmid detection and assembly in genomic and metagenomic data sets. Genome Res.

[19] Meyer F, Paarmann D, D’Souza M, Olson R, Glass EM, Kubal M (2008). The metagenomics RAST server—a public resource for the automatic phylogenetic and functional analysis of metagenomes. BMC Bioinformatics.

[20] Sentchilo V, Mayer AP, Guy L, Miyazaki R, Tringe SG, Barry K (2013). Community-wide plasmid gene mobilization and selection. ISME J.

[21] Camacho C, Coulouris G, Avagyan V, Ma N, Papadopoulos J, Bealer K (2009). BLAST+: architecture and applications. BMC Bioinformatics.

[22] Gweon HS, Shaw LP, Swann J, De Maio N, Abuoun M, Niehus R (2019). The impact of sequencing depth on the inferred taxonomic composition and AMR gene content of metagenomic samples. Environ Microbiomes.

[23] Cock PJA, Antao T, Chang JT, Chapman BA, Cox CJ, Dalke A (2009). Biopython: freely available Python tools for computational molecular biology and bioinformatics. Bioinformatics.

[24] Nishida H (2012). Evolution of genome base composition and genome size in bacteria. Front Microbiol.

[25] Ali J, Khan R, Ahmad N, Maqsood I. Random Forests and Decision Trees. 2012. www.IJCSI.org. Accessed 25 Aug 2020.

[26] Pedregosa F, Michel V, Grisel O, Blondel M, Prettenhofer P, Weiss R, et al. Scikit-learn: Machine Learning in Python. 2011. http://scikit-learn.sourceforge.net. Accessed 25 Aug 2020.

[27] Boughorbel S, Jarray F, El-Anbari M (2017). Optimal classifier for imbalanced data using Matthews Correlation Coefficient metric. PLoS ONE.

