# Supplementary material for: PlasForest: a homology-based random forest classifier for plasmid detection in genomic datasets
Source: BMC Bioinformatics. 2021 Jun 26;22:349. doi: 10.1186/s12859-021-04270-w (PMC8236179; doi:10.1186/s12859-021-04270-w)
Supplement: Supplementary file 1 — Additional file 1. Figure S1: Compared running time and memory use for PlasForest and PlasClass. [file 12859_2021_4270_MOESM1_ESM.docx]

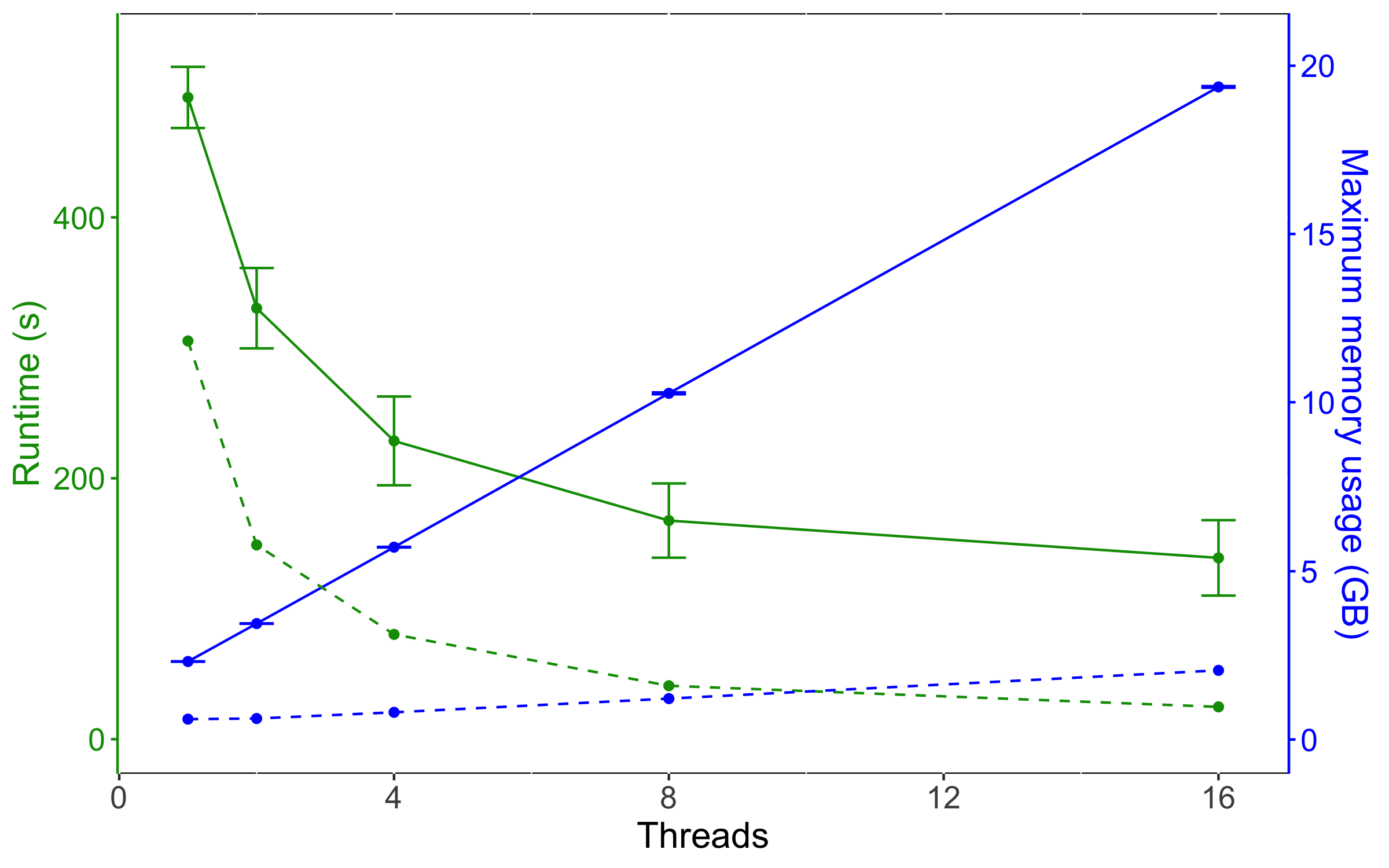


**Figure S1: Runtime and memory usage of the PlasForest pipeline.** PlasForest was run 10 times against a random sample of 1000 contigs from the COMGENOME dataset, with different number of threads. Runtime in seconds is displayed in green points and lines, maximum memory usage is displayed in blue points and lines. Error bars represent the standard deviation on runtime and maximum memory usage. The performances of PlasClass on the same dataset are displayed in dotted lines for comparison.
